# Hybridization–exchange interplay governs defect tolerance in halide perovskites

**DOI:** 10.1039/d6ra04433f

**Published:** 2026-09-01

**Authors:** Misbah Shaheen, Sheharyar Pervez

**Affiliations:** a Ghulam Ishaq Khan Institute of Engineering Sciences and Technology Pakistan sheharyar@giki.edu.pk

## Abstract

The role of partially filled 3d states in governing defect tolerance in halide perovskites remains unclear, particularly in the presence of strong intra-atomic exchange and metal-halide covalency. Using first-principles calculations on substitutional 3d transition metals in CsPbI_3_, we show that defect tolerance is not governed by the strength of d–p hybridization alone, but by its interplay with exchange splitting and crystal-field alignment. Early 3d dopants illustrate this balance: insufficient hybridization combined with low filling promotes localized deep states, whereas an optimal hybridization window stabilizes resonant states. In contrast, increasing d-shell occupancy enhances intra-atomic exchange, progressively localizing spin density and driving a crossover to exchange-dominated behavior in the late 3d regime. The resulting defect character reflects a competition between covalent delocalization and exchange stabilization, which together determine orbital ordering, magnetic polarization, and the spatial extent of defect states. These results establish a chemically transparent descriptor framework based on hybridization–exchange balance for integrating spin-active dopants without necessarily compromising electronic quality.

## Abstract 2

First-principles calculations show that defect tolerance in 3d-doped halide perovskites is governed by metal–halide hybridization and intra-atomic exchange, guiding the design of spin-active dopants.

## Graphical abstract

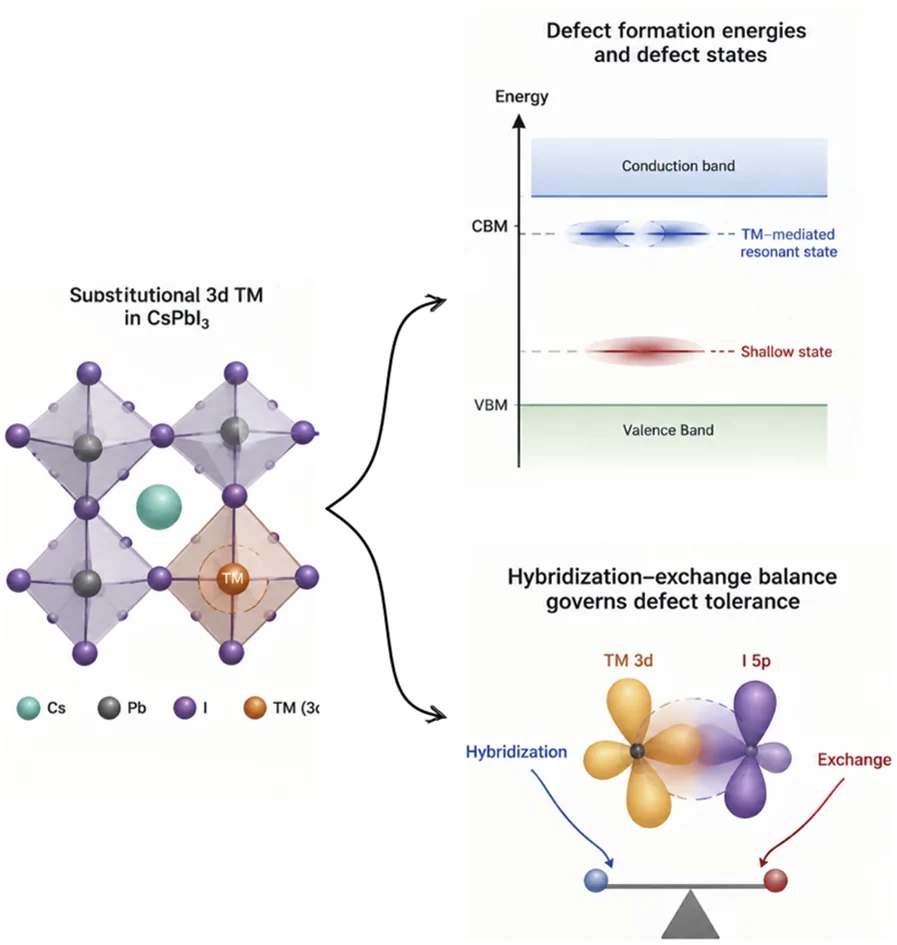

## 1. Introduction

Though the concept can be elusive and while competing definitions exist,^1–3^ inorganic halide perovskites (IHPs) are renowned for their intrinsic defect tolerance, which underpins their exceptional optoelectronic performance and photovoltaic appeal.^4–7^ Extrinsic transition-metal (TM) dopants offer a pathway to functionalize these materials with catalytic, magnetic, and optical or electronic properties, yet the impact of these partially filled d states on defect energetics and system chemistry remains poorly understood.^8,9^ While shallow states introducing free carriers into the system serve as the usual and conventional purpose of doping; certain dopants could introduce deep-level traps that may act as non-radiative recombination centers which undermine the host's transport capabilities as well as defect tolerance. These traps can reduce the minority carrier lifetime, decrease the open-circuit voltage (*V*_oc_), and limit the overall power conversion efficiency (PCE) of the solar cell.^10–14^ On the other hand, defects whose states lie outside the band gap or are successfully passivated may render the material more robust against electronic degradation. Since such states are typically fully occupied (in the valence band (VB)) or empty (in the conduction band (CB)), and therefore do not function as active carrier traps,^15–19^ the defect is electronically benign and can even contribute to thermodynamic stability, due to hybridization with bulk states. Identifying the exact types of defects present under varying synthesis and operating conditions,^20^ and correlating them reliably with their associated trap states,^21^ continues to be an empirical challenge. Theoretical work has systematically examined intrinsic defect formation energies and charge-state stability in CsPbX_3_ compounds, revealing a pronounced halide dependence of defect energetics that strongly affects carrier trapping behavior and material stability;^22^ similar work on extrinsic transition metal defects is lacking.

This study takes a first-principles approach to answer a central question: how do partially filled 3d shells, with their strong intra-atomic exchange and variable metal–halide hybridization, influence defect tolerance in halide perovskites? Our results indicate that defect tolerance is not determined by the magnitude of d–p hybridization alone, but by its interplay with d-shell filling, exchange splitting, and crystal-field alignment. This competition produces distinct regimes across the 3d series, ranging from hybridization-stabilized resonant states to exchange-driven localized defects. The rest of the study is organized as follows: Section 1 lays out the theoretical background, Section 2 contains the methodology; Section 3 carries results and discussions, wherein we link localized lattice distortions with electronic hybridization and spin polarization effects, to develop a consistent physical picture of how resonant and deep defect states emerge and elicit general design principles; and finally concluding remarks are presented in Section 4.

## 2. Preliminaries

Dopants induce defects in semiconductors and photovoltaic (PV) materials which create new electronic states within the electronic band structure. These defects, broadly classified in Table 1, may be deep/trap (inside bandgap far from edges), shallow (inside bandgap near the valence or conduction band) or resonant (inside VBM or CBM). On the whole, trap states are regarded as detrimental to photovoltaic efficiency because they act as recombination centers that limit carrier lifetime and voltage. Although intermediate-band solar cells aim deliberately engineered trap-like states that allow absorption of sub-bandgap photons, potentially enhancing photocurrent; achieving this effect without introducing significant recombination losses remains a challenge. A careful understanding of both the energetic position and the chemical passivation of trap states is thus central to the design of next-generation, stable, and efficient photovoltaic devices.

**Table 1:** Classification of defect states by type and energetic position

Defect	Inside bandgap	Merges with VBM/CBM	Carrier trapping and recombination	Acceptors or donors
Resonant	✗	✓	✗	✓
Deep	✓	✗	✓	✓
Shallow	✓	✗	✗	✓

### 2.1. Defect formation energies (DFEs)

Thermodynamically, DFEs describe the ease with which a certain defect can be formed. For the case of a defect *X* with charge *q* in a host structure, this can be written as:1
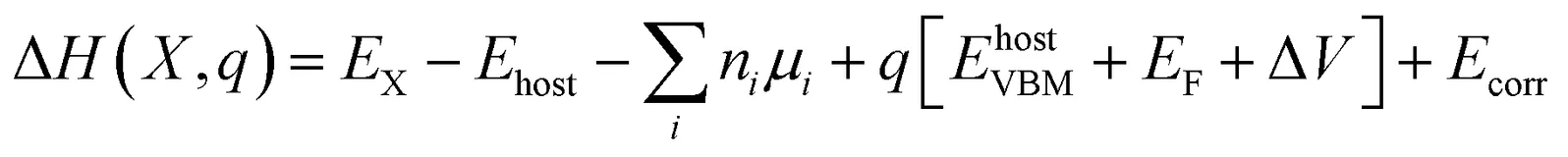
where *E*_X_ and *E*_host_ represent the total energy of defected and pristine structures, respectively. *n*_*i*_ is the number of atoms added (*n*_*i*_ > 0) or removed (*n*_*i*_ < 0) from the system for which the energy *µ*_*i*_ is required. The charge of the defect *q* is the number of electrons transferred to or from the reservoir to create a defect, *E*_VBM_ is the valence band maximum (VBM) of the host material, and *E*_F_ is the Fermi level (electron chemical potential), referenced to the valence band maximum (VBM), which determines the energy required to add or remove electrons when forming a charged defect. *E*_corr_ is the energy correction. Determining the VBM in defective systems is tricky because defects perturb the potential energy surface by introducing states inside the bandgap. Thus, the VBM of the pristine structure is usually taken as the reference point, albeit shifted by a potential alignment term Δ*V*, calculated by comparing the core level energy of pristine and defective systems. *E*_F_ then is the energy added on top of the reference point *E*_VBM_ + Δ*V* and usually taken as zero at VBM.

Charge state transition level (CSTL)—a charge state transition occurs when the defect formation energies of two charge states, *q* and *q*′, become equal. The corresponding Fermi level is known as the charge state transition level (CSTL). CSTLs are a useful indicator of whether the new energy state is a detrimental trap or a useful energy state and are calculated using:^23^2
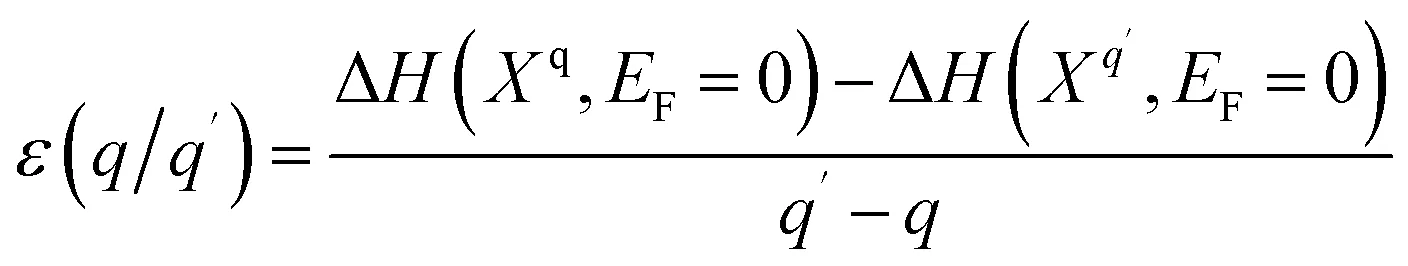
Here, Δ*H* shows the DFE of charge state *q* or *q*′ at VBM *i.e. E*_F_ = 0.

Energy Corrections—DFT codes frequently employ periodic boundary conditions which mandates the use of the supercell method to isolate the defect and prevent the interactions between periodic images. For charged defects, the long range nature of the Coulomb interaction then yields divergent electrostatic behavior. To avoid using a very large supercell, *E*_corr_ is the correction needed to eliminate the effects of electrostatic interactions between charged defects in periodic images.^24,25^

The charge correction scheme used here, was introduced by Freysoldt, Neugebauer, and Van de Walle (FNV)^24^ and requires the alignment of the potentials of charged and neutral defected supercells due to the undefined reference electrostatic potential. The term *E*_corr_ in eqn (1) then takes the general form:

3 *E*_corr_ = *E*^lat^_*q*_ − *qΔ*_*q*/0_

where *E*^lat^_q_, the macroscopically screened lattice energy of defect charge with compensating background, accounts for the long range interactions and *qΔ*_*q*/0_ is the alignment term.

### 2.2. Phase stability

The range of chemical potentials (CPs) is thermodynamically constrained by the competing phases surrounding the region in which the host material CsPbI_3_ is the most stable phase. These constraints depend on the growth conditions and must be determined by keeping the stable region of host material in view. Thus the elemental chemical potentials are constrained by the formation enthalpy Δ*H* of CsPbI_3_, such that:

4 Δ*µ*(Cs) + Δ*µ*(Pb) + 3Δ*µ*(I) = Δ*H*(CsPbI_3_)

where Δ*µ* are the effective atomic CPs of the associated element in the perovskite environment with reference to the standard elemental phase. The remaining constraints prevent the formation of secondary phases (PbI_2_, Cs_4_PbI_6_, CsI_3_, and Pb) are:5
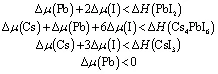


**Fig. 1:**
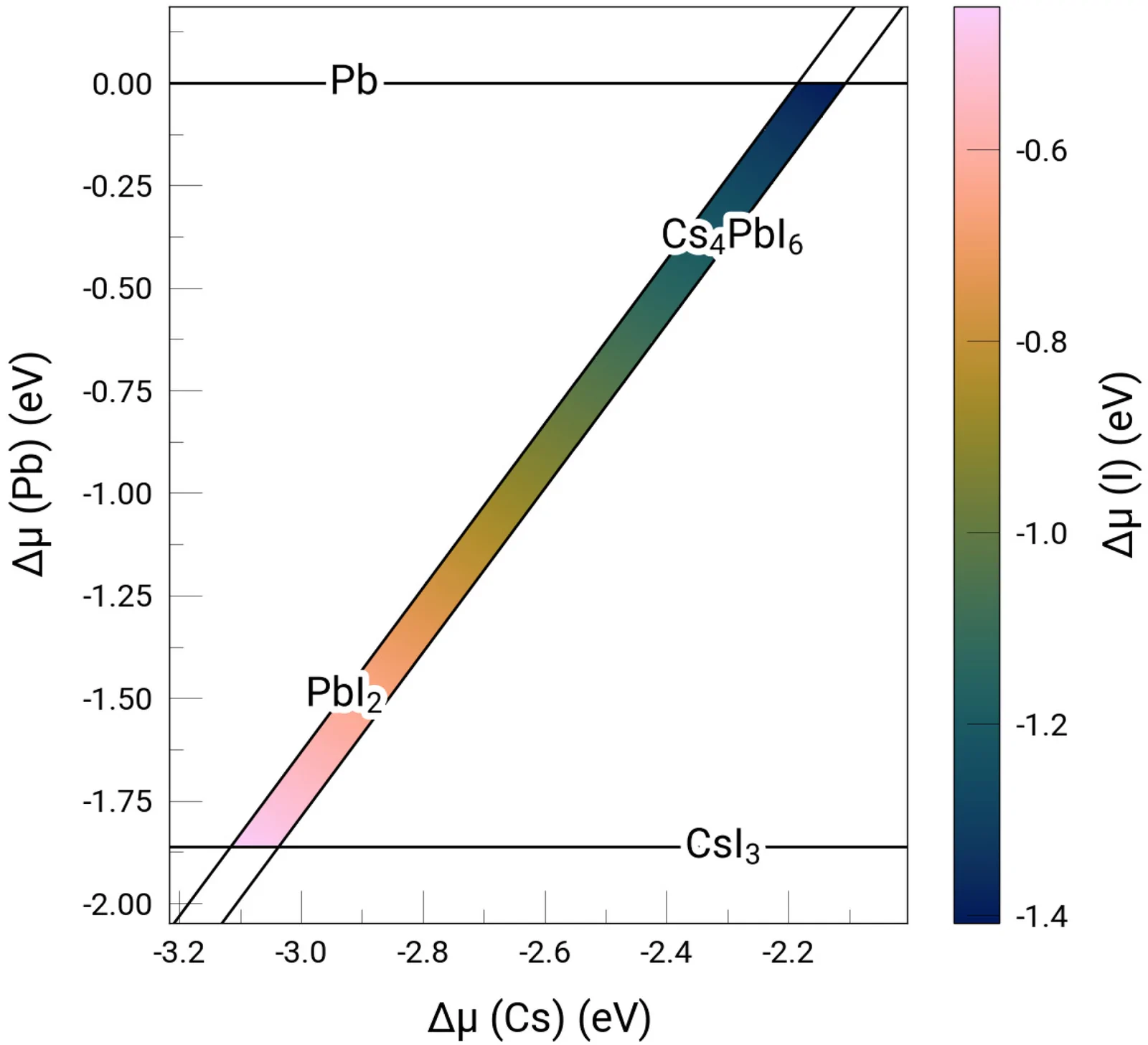
Stability polygon showing the stable region for CsPbI_3_ enclosed by competing phases.

Eqn (5) defines the stability polygon shown in Fig. 1. Pink region of the polygon shows the halogen rich end and the blue region is the halogen poor end. The conditions on CPs to obtain stable CsPbI_3_ in equilibrium with PBI_2_ and Cs_4_PbI_6_ in I-rich and I-poor environment are given in Table 2.

**Table 2:** Chemical potentials for elemental species present in CsPbI_3_ in equilibrium with PbI_2_ and Cs_4_PbI_6_ for I-rich and I-poor conditions

	I-rich	I-poor
Equilibrium with PbI_2_	Δ*µ*(I) = ½Δ*µ*(I^mol^_2_)	Δ*µ*(I) = ½[Δ*µ*(PbI_2_) − Δ*µ*(Pb^bulk^)]
	Δ*µ*(Pb) = Δ*µ*(PbI_2_) − Δ*µ*(I^mol^_2_)	Δ*µ*(Pb) = Δ*µ*(Pb^bulk^)
	Δ*µ*(Cs) = Δ*H*(CsPbI_3_) − Δ*µ*(PbI_2_) − ½Δ*µ*(I^mol^_2_)	Δ*µ*(Cs) = Δ*H*(CsPbI_3_) + ½Δ*µ*(Pb^bulk^) − <svg xmlns="http://www.w3.org/2000/svg" version="1.0" width="18.545455pt" height="16.000000pt" viewBox="0 0 18.545455 16.000000" preserveAspectRatio="xMidYMid meet"><metadata> Created by potrace 1.16, written by Peter Selinger 2001-2019 </metadata><g transform="translate(1.000000,15.000000) scale(0.015909,-0.015909)" fill="currentColor" stroke="none"><path d="M80 840 l0 -40 -40 0 -40 0 0 -40 0 -40 40 0 40 0 0 40 0 40 120 0 120 0 0 -40 0 -40 -80 0 -80 0 0 -40 0 -40 80 0 80 0 0 -80 0 -80 -120 0 -120 0 0 40 0 40 -40 0 -40 0 0 -40 0 -40 40 0 40 0 0 -40 0 -40 120 0 120 0 0 40 0 40 40 0 40 0 0 80 0 80 -40 0 -40 0 0 40 0 40 40 0 40 0 0 40 0 40 -40 0 -40 0 0 40 0 40 -120 0 -120 0 0 -40z M720 840 l0 -40 -40 0 -40 0 0 -80 0 -80 -40 0 -40 0 0 -80 0 -80 -40 0 -40 0 0 -80 0 -80 -40 0 -40 0 0 -80 0 -80 -40 0 -40 0 0 -40 0 -40 -40 0 -40 0 0 -40 0 -40 80 0 80 0 0 40 0 40 40 0 40 0 0 80 0 80 40 0 40 0 0 80 0 80 40 0 40 0 0 -40 0 -40 80 0 80 0 0 40 0 40 40 0 40 0 0 -80 0 -80 -40 0 -40 0 0 -40 0 -40 -40 0 -40 0 0 -40 0 -40 -40 0 -40 0 0 -40 0 -40 200 0 200 0 0 40 0 40 -80 0 -80 0 0 40 0 40 40 0 40 0 0 80 0 80 40 0 40 0 0 40 0 40 -40 0 -40 0 0 40 0 40 -120 0 -120 0 0 -40 0 -40 -40 0 -40 0 0 80 0 80 40 0 40 0 0 80 0 80 40 0 40 0 0 80 0 80 -40 0 -40 0 0 -40z"/></g></svg> Δ*µ*(PbI_2_)
Equilibrium with Cs_4_PbI_6_	Δ*µ*(I) = ½Δ*µ*(I^mol^_2_)	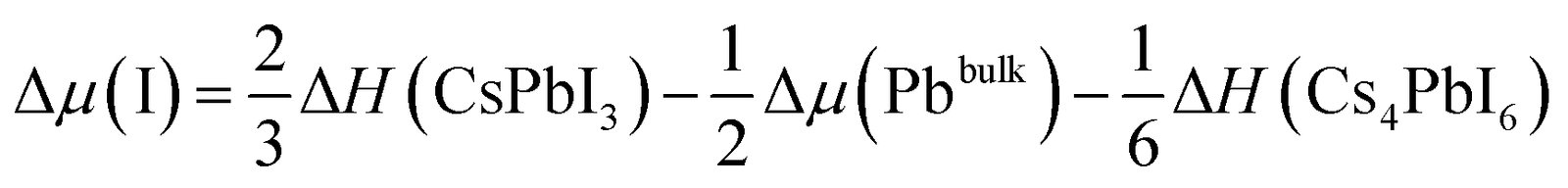
	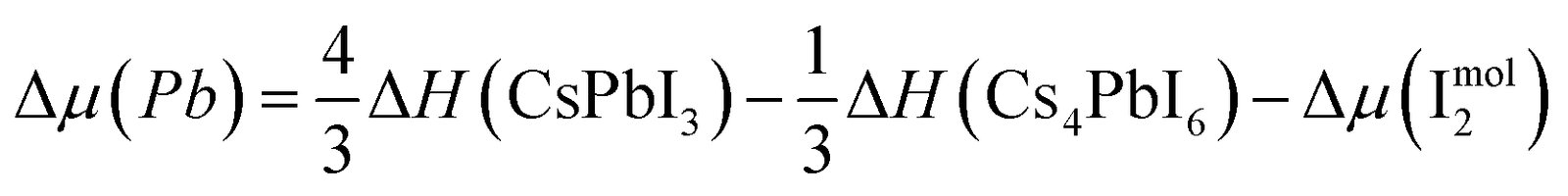	Δ*µ*(Pb) = Δ*µ*(Pb^bulk^)
	Δ*µ*(Cs) = ⅓[Δ*H*(Cs_4_PbI_6_) − Δ*H*(CsPbI_3_)] − ½Δ*µ*(I^mol^_2_)	Δ*µ*(Cs) = ½Δ*H*(Cs_4_PbI_6_) − Δ*H*(CsPbI_3_) + ½Δ*µ*(Pb^bulk^)

### 2.3. Octahedral distortions

Displacement of ligands coordinated with the central atom distorts the octahedron and can be described through Van Vleck modes.^26,27^

Bond-length distortion modes relevant to this study are (i) the isotropic breathing mode Q_1_, which describes the uniform expansion/contraction of the octahedron, and (ii) the tetragonal mode Q_3_, where axial and equatorial ligands behave differently, resulting in a compressed or elongated octahedron. The magnitude of distortion, *ρ*_0_, for the Q_3_ mode is given by:6
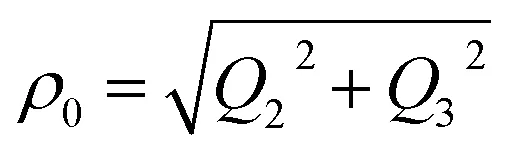
where the magnitude is determined using the basis vectors *Q*_2_ and *Q*_3_.

Bond-length distortion index is defined by Baur^28^ as:7
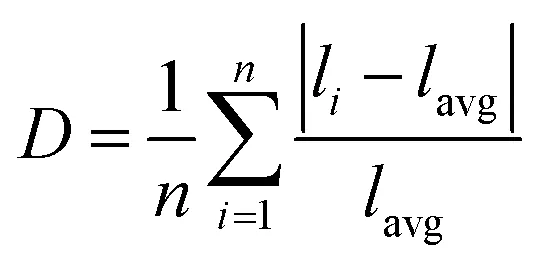
where *n* is the number of ligands, *l*_*i*_ being the distance between central atom and the *i*-th ligand, and *l*_avg_ is the average of all distances between central atom and ligands.

Effective coordination number (ECN)^29^ is another parameter which describes the deviation from the regular octahedral geometry:8
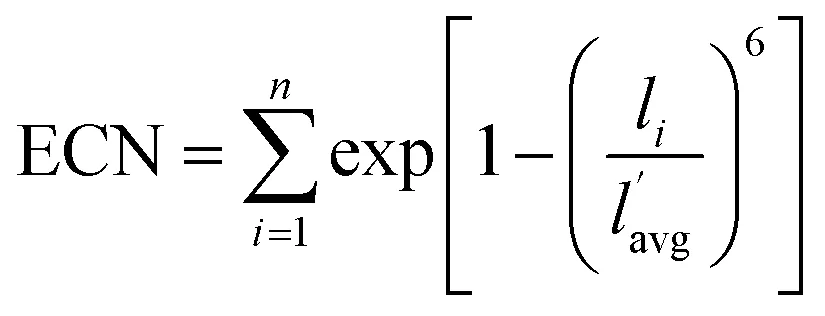
Here, 
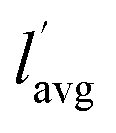
 is the modified average of distances which is equal to:9
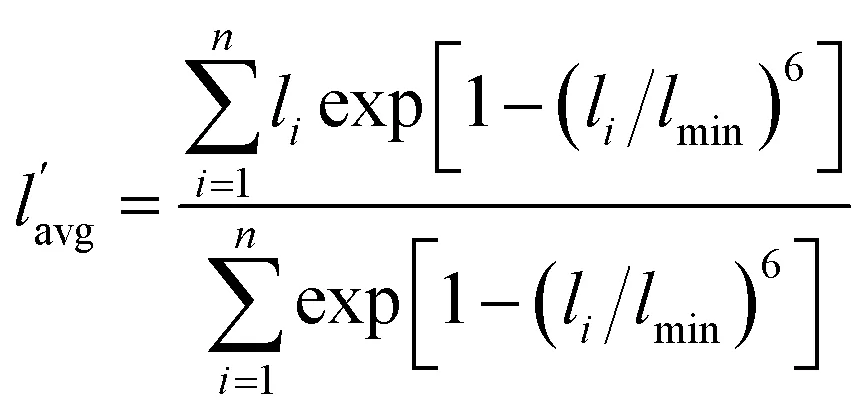


## 3. Methodology

**Fig. 2:**
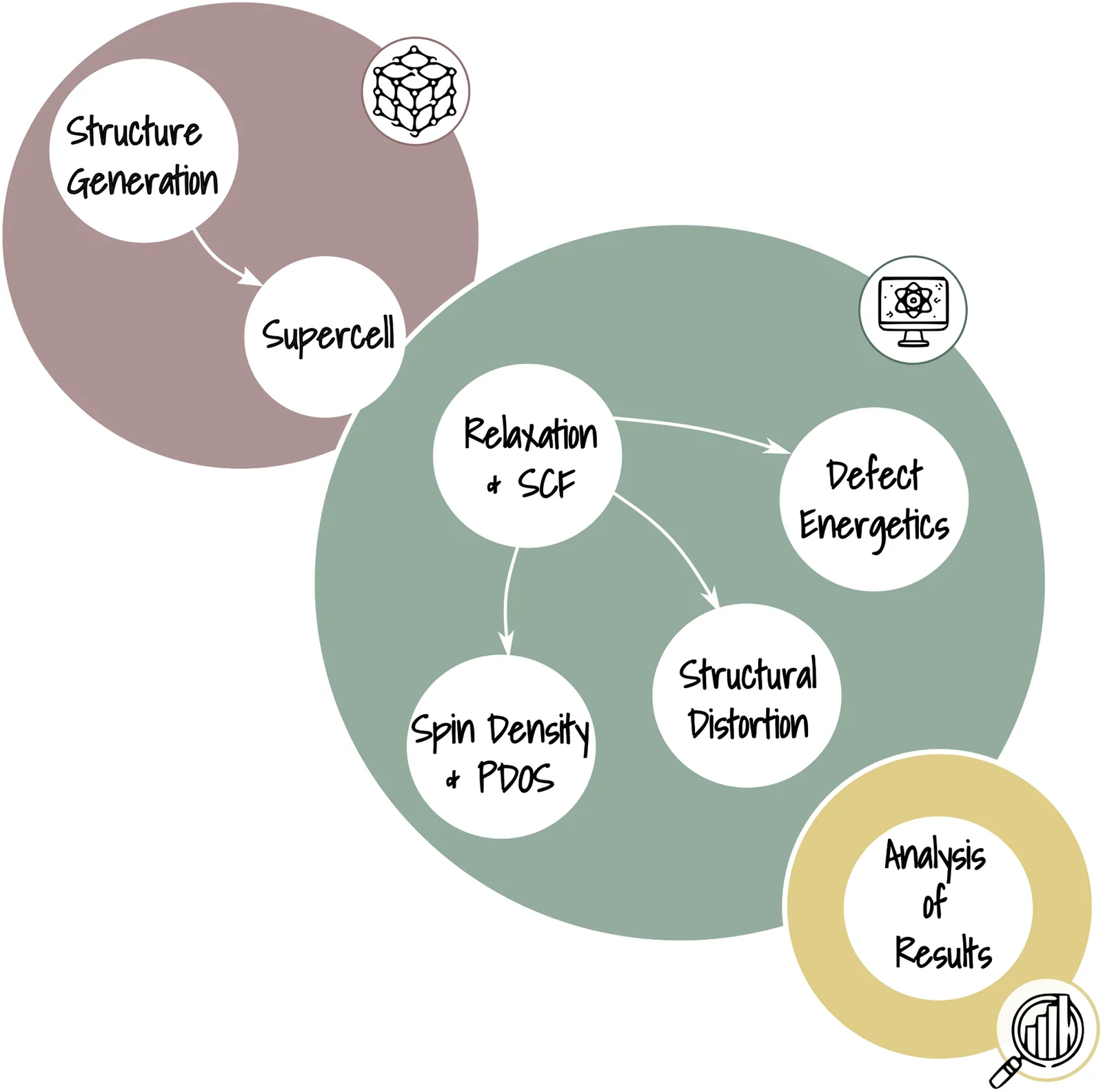
Aspects of the adopted workflow. The generated structures were evaluated on defect energetics, structural distortion and spin behavior.

The key elements of our workflow are summarized in Fig. 2.

Defected structures were generated using 
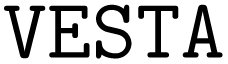
^30^ by taking a 2 × 2 × 2 supercell of the host material, CsPbI_3_ and replacing Pb at the origin with 3d-TMs (Sc, Ti, V, Cr, Mn, Fe, Co, Ni) one by one as point defects in the crystal. DFT calculations were performed in a plane-wave basis set using Quantum Espresso v.7.4 (QE)^31^ with ultrasoft pseudopotentials obtained from PSLibrary and the PBE exchange correlation functionals^32^ to optimize the geometry and obtain energies of bulk and defected structures. The host supercell was fully relaxed as bulk, and then used as the template for defected structures. Parameters and thresholds used for DFT calculations are given in Table S2. Relaxations of defected structures, self consistent field (SCF), and partial density of states (PDOS) calculations of doped structures were performed using QE with Hubbard correction within the spin-polarized GGA+*U* framework (Table S3). Goldschmidt tolerance (*t*) and octahedral factor (*µ*) were computed to assess the structural stability (see Section S1). Another tolerance factor was calculated proposed by Bartel *et al.*^33^ to estimate the perovskite stability (S1). Materials project^34^ and python libraries including 
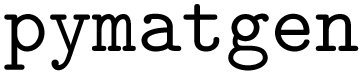
^35^ and 
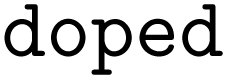
^36^ were used for defect calculations to obtain defect formation energy (DFE), charge state transition levels (CSTLs), competing phases, and Freysoldt energy corrections (see Section S1 for details). The functionality of 
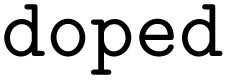
 was expanded to add support for QE and is available on its github repository. Relaxed doped structures were further analyzed using the python package 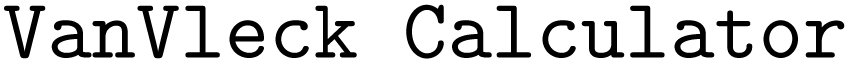
^37^ and 
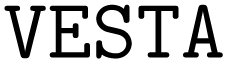
 to determine the distortion parameters (Section IC). Spin densities were computed using the post-processing modules provided with QE. Crystal field, exchange splittings and orbital overlaps were computed by estimating the orbital centroids of corresponding orbitals manifold using PDOS (Section S1C).

## 4. Results and discussion

**Fig. 3:**
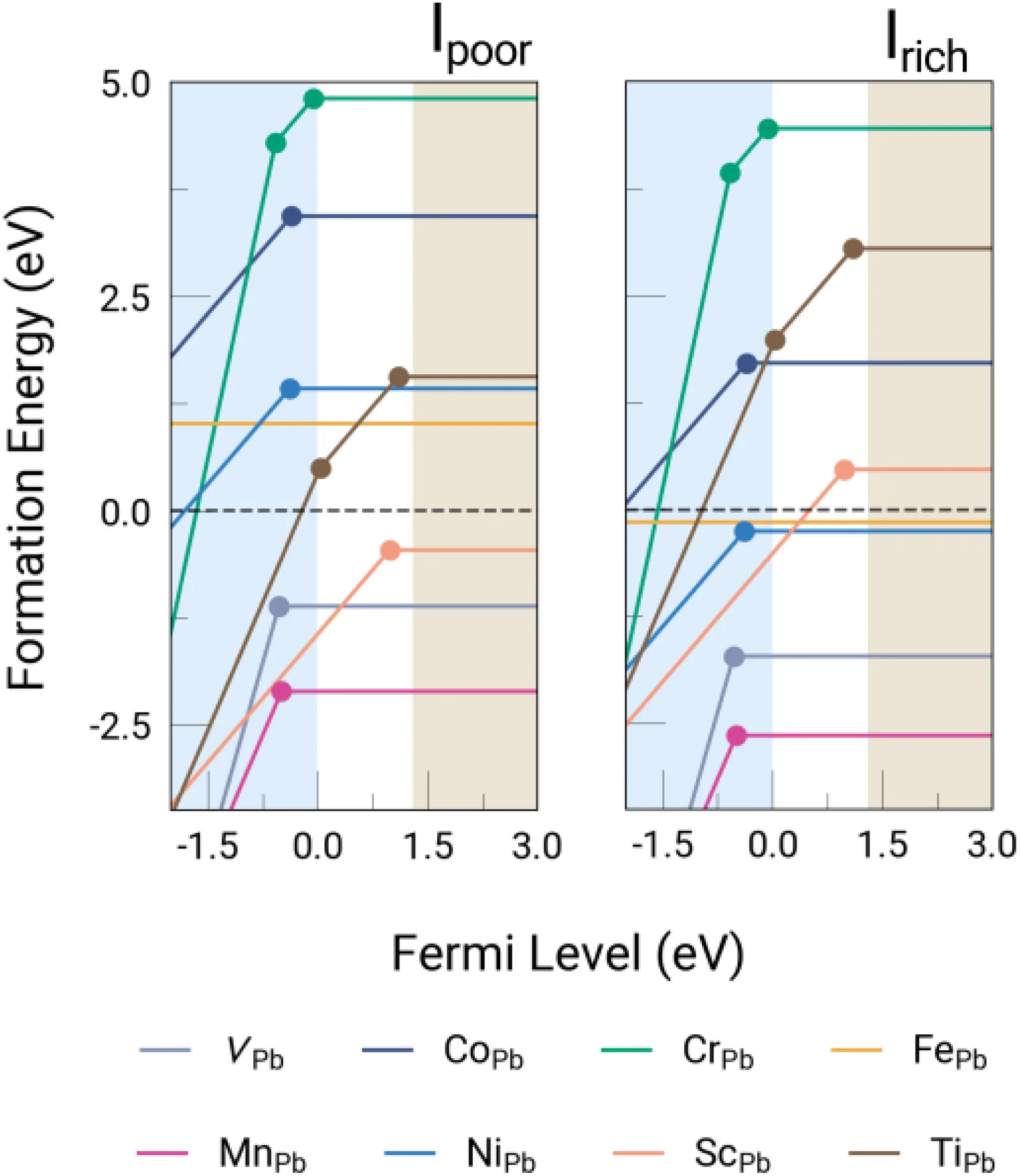
Defect formation energies in I-poor (left) and I-rich (right) show Mn as the most stable defect and Cr the least. Only Sc and Ti form deep traps.

We begin our analysis by looking at defect thermodynamics and the DFEs of these substitutional 3d dopants. Fig. 3 reveals that most defects remain neutral within the bandgap and are preferentially stabilized under I-rich conditions; with the exception of Sc and Ti which uniquely exhibit +3/+2 charge-state transition levels and are preferentially stabilized under I-poor conditions (Table S5).

**Fig. 4:**
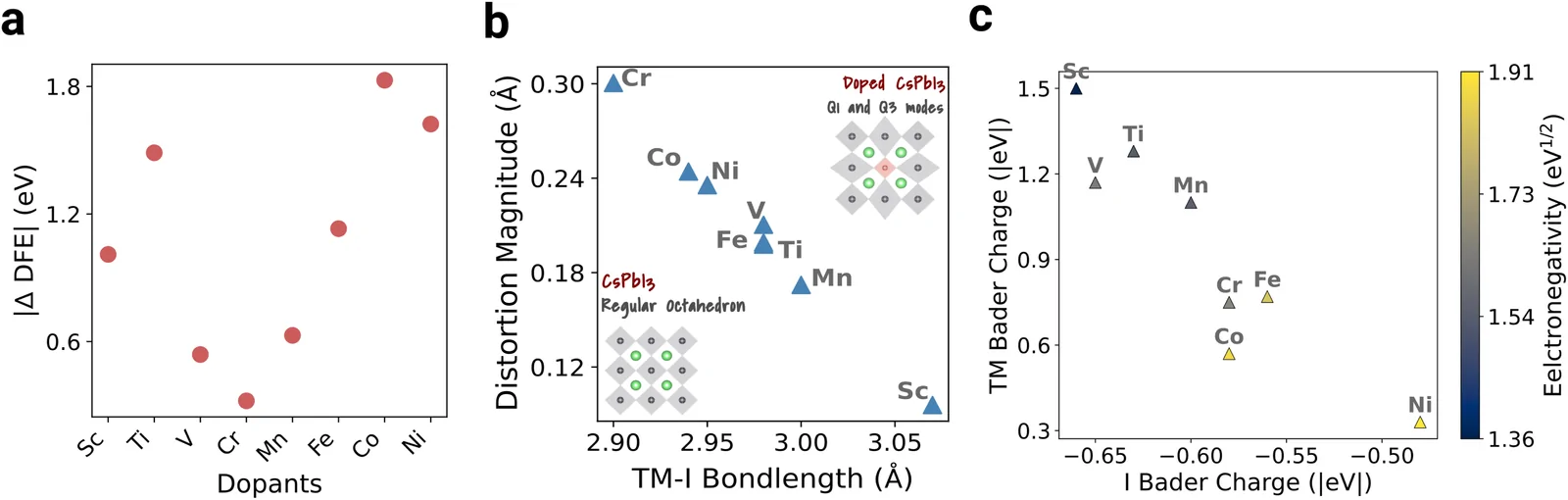
(a) Sensitivity of defects to I growth conditions measured in terms of the difference of DFE across I-rich and I-poor environments. (b) Distortion magnitude of the PbI_6_ octahedron adjacent to TM octahedron *vs.* TM–I bond length. (Insets) Bottom Left: Schematic showing the regular octahedrons in pristine CsPbI_3_. Top right: Shrinked TM octahedron and distorted PbI_6_. (c) Bader charge analysis between TM and I. Top left indicates less covalency.

The DFE decreases monotonically across all TM species with decreasing oxidation state, consistent with the concomitant increase in Shannon ionic radius (Fig. S1, S2, and Table S4), indicating that lower-valent, larger dopants impose weaker lattice perturbations. Mn is consistently the most stable defect, and along with V shows moderate sensitivity to growth conditions. Cr, on the other hand, exhibits the highest formation energies across growth conditions and is largely insensitive to them (Fig. 4(a)).

These defects introduce local strain-driven distortions which relieve lattice stress ensuring structural stability. The distortions in the local environments stem from the strain caused by the Van Vleck modes Q_1_ and Q_3_ for the TM and its adjacent PbI_6_ octahedron respectively (Fig. 4(b)). But while the dopant octahedra shrinks symmetrically, due to the smaller ionic radii, partially filled d-orbitals, and stronger electron sharing with p-orbitals, the adjacent Pb octahedron is stretched asymmetrically.

Within the 2 × 2 × 2 supercell considered here, the structural distortion is primarily localized around the substituted TMX_6_ octahedron. The largest bond-length and octahedral distortions occur at the dopant site, while the adjacent PbX_6_ octahedra exhibit smaller distortions. Octahedra beyond the first coordination shell remain same as their pristine geometry with zero distortion as per the Van Vleck distortion analysis, indicating that the perturbation is highly localized within the simulated supercell.

The linear scaling between distortion parameters and TM–I bond length indicates that local structural distortion is primarily governed by size-mismatch-induced strain, with Cr exhibiting the strongest distortion due to its shortest TM–I bond length, and resulting in the smallest coordination number for the Pb atom in the adjacent octahedra (Fig. S3 and Table S6).

The shortening of the TM–I bond relative to the 3.15 Å Pb–I bond primarily originates from the smaller ionic radii of the substituted transition metals. The equilibrium bond length is further influenced by the degree of TM–I hybridization as well as crystal-field and exchange effects for specific d-electron configurations. Owing to the high polarizability of iodine, the extent of charge transfer varies across the transition-metal series. As shown by the Bader charge analysis in Fig. 4(c), early TMs (Sc, Ti, and V) donate more charge to iodine, resulting in predominantly ionic TM–I bonding, whereas late TMs (Co and Ni) exhibit reduced charge transfer and stronger TM d–I p hybridization, leading to more covalent interactions.

**Fig. 5:**
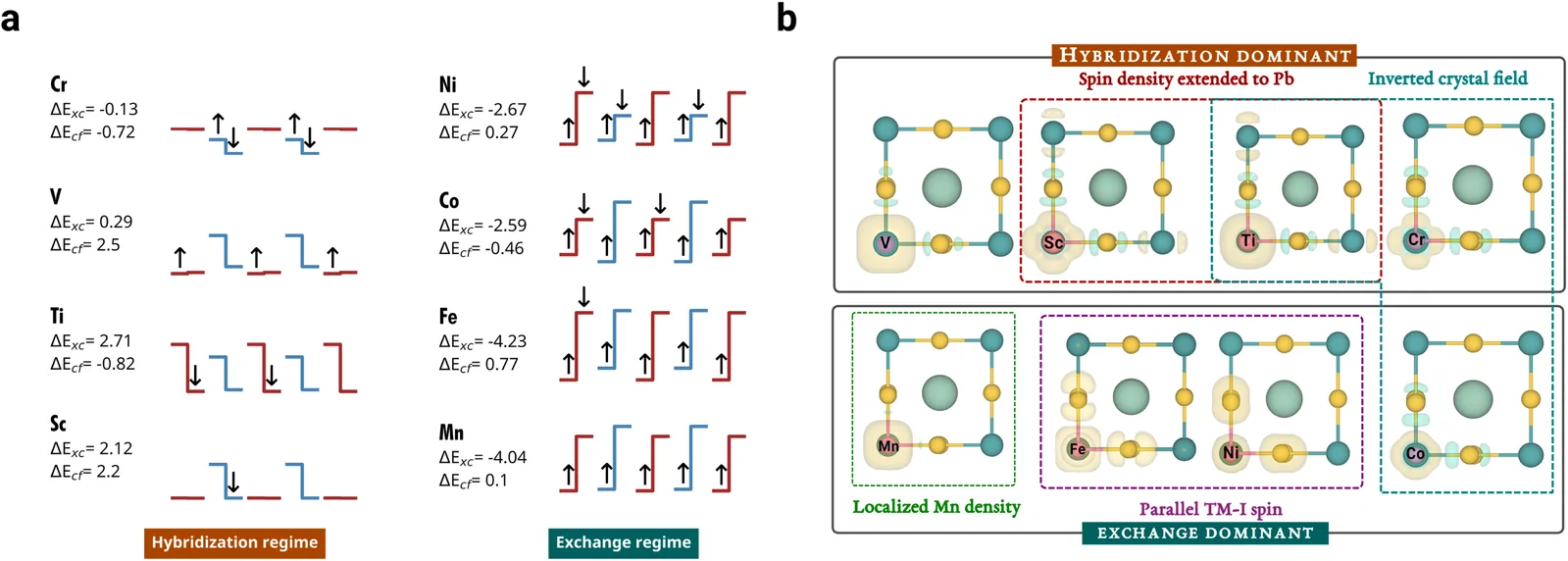
(a) Orbital-resolved centroids of the crystal-field and exchange splittings for early and late transition metals, shown for the e_g_ (blue) and t_2g_ (red) manifolds. An inverted crystal-field ordering is observed for Cr, Co, and Ti. Late transition metals exhibit pronounced exchange splitting compared to their early counterparts. (b) Spin-density isosurfaces showing positive (light yellow) and negative (light blue) polarization. Atomic species are indicated as TM (pink), I (gold), Pb (teal), and Cs (sage green). Early transition metals (top) exhibit a hybridization-dominated electronic regime, while late transition metals (bottom) display exchange-splitting-dominated behavior.

For certain metals, strong σ-type hybridization of e_g_ states with iodine p orbitals can overcompensate the electrostatic crystal-field splitting, lower the bonding e_g_–p manifold beneath t_2g_, and invert the conventional octahedral order. This inversion is strongest in Cr and moderate in Co and Ti (Fig. 5(a)). Since Cr also exhibits a collapsed exchange splitting (0.13 eV) it stabilizes into a low-spin configuration with 0.59*µ*_B_ magnetization (Table 3). Fig. 5(a) also shows that, in late TMs, exchange splitting dominates over crystal-field effects, favoring high-spin states and enhanced magnetic stabilization. Mn has the largest difference between the exchange and crystal field splittings (Table 3) and is the most stable defect. Fig. 5(b) shows the spin density distribution. The TM–I bond-aligned lobed profiles of Sc, Cr, and Co indicate significant e_g_ character and directional d–p coupling. In contrast, Mn, Fe, and Ni exhibit less directional spin textures, consistent with dominant t_2g_ occupation or stronger exchange-driven localization.

**Table 3:** Magnetic and electronic structure parameters of early and late transition metal (TM) dopants in CsPbI_3_, including total and absolute magnetization, d–p orbital overlap, exchange splitting (*Δ*_Ex_) of e_g_ and t_2g_ states, crystal-field (CF) splitting, and overall exchange energy

Group	TM	*M*_tot_ (*µ*_B_)	*M*_abs_ (*µ*_B_)	d–p overlap (eV^−1^)	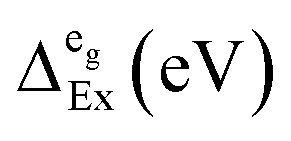	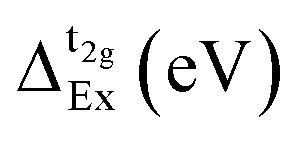	*Δ*_CF_ (eV)	*Δ* ^tot^_Ex_ (eV)
Early	Cr	0.59	1.21	0.09	0.91	0.03	−0.72	−0.13
	Sc	0.90	1.38	0.21	2.21	0.01	2.20	2.12
	Ti	1.96	2.45	0.07	2.15	3.07	−0.82	2.71
	V	3.00	3.42	0.69	2.04	−0.08	2.50	0.29
Late	Co	2.11	2.79	0.19	−3.90	−2.29	−0.46	−2.59
	Fe	4.71	4.89	0.02	−3.77	−4.39	0.77	−4.23
	Mn	5.00	5.12	0.02	−4.12	−3.56	0.10	−4.04
	Ni	2.28	2.34	0.03	−1.61	−3.38	0.27	−2.67

**Fig. 6:**
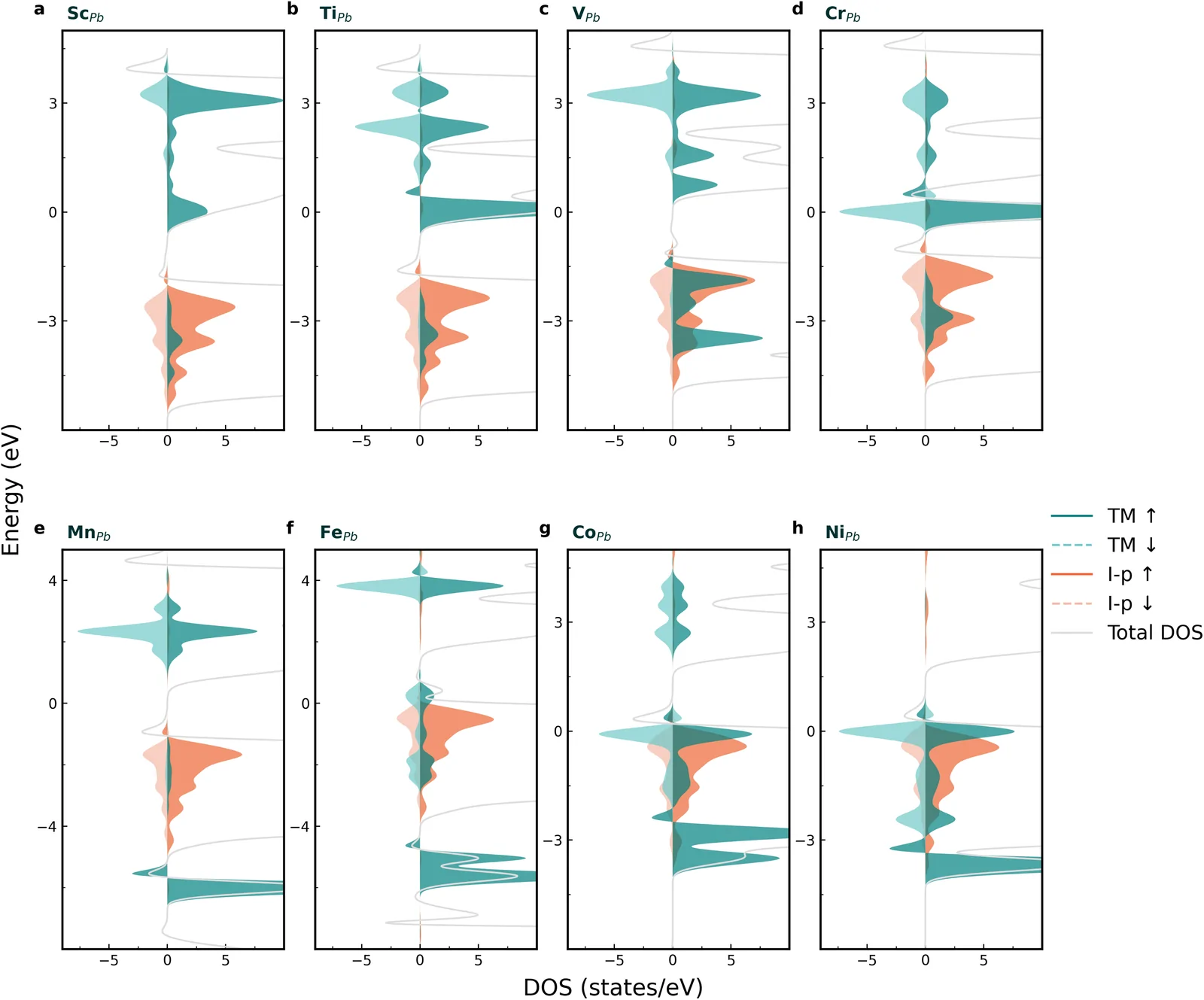
Spin-polarized partial density of states (PDOS). (a–d) Early transition metals display substantial unoccupied d-character in the conduction band and strong d–p hybridization in the valence. (e–h) For Mn and the subsequent late transition metals, the increased d-electron filling reduces the availability of unoccupied conduction-band d-states. Here, exchange splitting becomes the dominant interaction, outweighing hybridization effects.

**Fig. 7:**
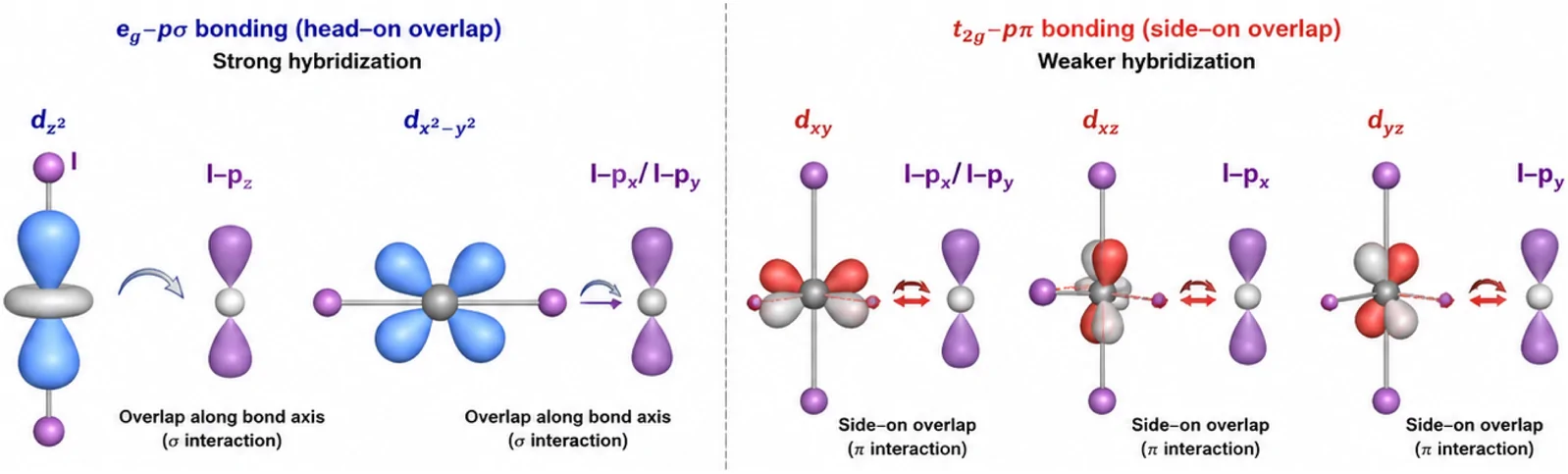
Schematic showing the bonding of I-p orbitals with e_g_ (sigma) and t_2g_ (pi) orbitals of TM.

Importantly, TM–I hybridization plays a dual electronic role. Delocalizing charge and spin density onto the host lattice can also broaden defect states and promote their interaction with band-edge states. When this hybridization is sufficiently strong and energetically aligned (as is the case for V), defect levels become resonant rather than deep mid-gap traps, thereby preserving electronic structure, as evidenced by the spin-polarized PDOS (Fig. 6). The PDOS figure also shows moderate to low overlap for the late TMs. Orbital-resolved PDOS analysis shows that the overlap between the TM e_g_ states and I-p orbitals exceeds that of the t_2g_ states for all dopants except V (Table S6). Because the e_g_ orbitals point directly toward the iodine ligands, this enhanced overlap is characteristic of stronger σ-type TM–I hybridization, whereas the t_2g_ orbitals, oriented between the ligands, primarily contribute to weaker π-type interactions (Fig. 7).

The energy alignment mentioned above explains why V does not create deep traps, but Ti and Sc do. The enhanced stability of Sc and Ti under I-poor growth conditions reflects chemical potential constraints that favor their higher oxidation states. That same high-valent character also produces strong local electrostatic perturbations, giving rise to deep trap states that may act as recombination centers detrimental to optical performance. Additionally, V exhibits the largest d–p overlap (Table 3), substantially exceeding Sc and Ti. The relatively weak d-shell filling combined with insufficiently delocalized d–p coupling leads to localized, energetically isolated states that manifest as deep traps. In contrast, V-despite its large overlap-appears to reside in an electronically favorable regime where hybridization is strong enough to delocalize defect states toward the band edges without driving metallic behavior. Thus, V occupies an optimal hybridization window: strong enough to prevent localization, yet balanced by favorable d-shell occupancy.

Thus, for early TMs at least, defect tolerance is governed not by the magnitude of overlap, but by its interplay with electronic filling and level alignment. Excessively weak hybridization promotes localization (deep trapping), whereas sufficiently strong and well-aligned hybridization stabilizes resonant states. This reinforces the need for descriptor models that incorporate both hybridization strength and shell filling rather than relying on overlap integrals alone.

Increasing d-shell filling brings intra-atomic exchange splitting into the fray, progressively stabilizing local moments and suppressing long-range spin delocalization. This produces a crossover from hybridization-dominated donor-like behavior in early 3d substitutions to exchange-driven localization in the late 3d regime, as seen in the PDOS as well as exchange splitting (Table 3). V and Mn represent electronically stabilized boundary cases: V retains conventional ordering despite appreciable covalency, due to its small exchange splitting in the t_2g_ manifold, whereas Mn defines the strongly exchange-dominated limit with minimal hybridization, where the high-spin d^5^ configuration and large exchange splitting confine the spin density almost entirely to the TM site.

## 5. Conclusion

We have shown that the behavior of substitutional 3d dopants in CsPbI_3_ is governed by the balance between covalent TM–I d–p hybridization and intra-atomic exchange splitting. This competition controls the relative ordering of e_g_ and t_2g_ states, determines whether defect levels remain resonant with the band edges or form localized in-gap states, and sets the magnitude and spatial distribution of magnetic polarization. When hybridization dominates, orbital reordering and ligand-mediated spin delocalization emerge; when exchange prevails, high-spin configurations and enhanced energetic stabilization result. The resulting electronic structure and consequently defect stability is therefore not dictated by lattice distortion alone, but by the interplay between covalency and exchange that shapes orbital occupation, defect-state character, and magnetic texture across the dopant series.

## Conflicts of interest

There are no conflicts to declare.

## Supplementary Material

RA-016-D6RA04433F-s001

## Data Availability

Data for this article are available at https://zenodo.org/records/18335088. Supplementary information (SI) is available. See DOI: https://doi.org/10.1039/d6ra04433f.
