# Supplementary material for: Hybridization–exchange interplay governs defect tolerance in halide perovskites
Source: RSC Adv. 2026 Sep 1;16(42):51480–8. doi: 10.1039/d6ra04433f (PMC13531524; doi:10.1039/d6ra04433f)
Supplement: Supplementary file 1 [file RA-016-D6RA04433F-s001.pdf]

Supplementary Information (SI) for RSC Advances.
This journal is © The Royal Society of Chemistry 2026

Supplementary Information

Hybridization–Exchange Interplay Governs Defect Tolerance in Halide Perovskites

Misbah Shaheen and Sheharyar Pervez*
Ghulam Ishaq Khan Institute of Engineering Sciences and Technology

S1. METHODOLOGY

A. Goldschmidt tolerance and Octahedral factor

Goldschmidt tolerance factor (t) [1] octahedral factor (μ) were calculated using the equations below:

$$t = \frac{r_A + r_X}{\sqrt{2}(r_{B_{eff}} + r_X)} \tag{S1}$$

$$\mu = \frac{r_{B_{eff}}}{r_X} \tag{S2}$$

where, r_A is the radius of Cs, $r_{B_{eff}}$ is the effective radius of atoms on B-site including Pb and the defect, and r_X is the I radius. Effective radius for B-site is equal to $(7 \times r_B + r_{defect})/8.0$.

B. Tolerance Factor Proposed By Bartel et al. (τ)

In addition to the conventional Goldschmidt tolerance factor, the recently proposed Bartel tolerance factor (τ) [**?**] was evaluated to further assess the geometric stability of the investigated halide perovskites. Unlike the Goldschmidt tolerance factor, τ provides a probabilistic descriptor of perovskite stability and has been shown to exhibit improved predictive accuracy for both oxide and halide perovskites. According to Bartel *et al.*, compounds with $\tau < 4.18$ are generally expected to adopt a perovskite structure, while increasing values of τ correspond to a decreasing probability of perovskite stability.

The tolerance factor is calculated as:

$$\tau = \frac{r_X}{r_B} - n_A \left(n_A - \frac{\frac{r_A}{r_B}}{\ln\left(\frac{r_A}{r_B}\right)} \right), \tag{S3}$$

where r_A, r_B, and r_X are the Shannon ionic radii of the A, B, and X ions, respectively, and n_A is the oxidation state of the A-site cation. The Shannon ionic radii employed in this work are identical to those used for the Goldschmidt tolerance factor calculations.

The calculated τ values lie in the range of 4.18–4.23, placing the investigated compounds close to the empirical decision boundary ($\tau \leq 4.18$). As emphasized by Bartel et al., τ should be interpreted probabilistically rather than as a strict binary criterion, and compounds near the decision boundary cannot be confidently classified as stable or unstable solely on the basis of τ. Therefore, the obtained values indicate that the TM-substituted halide perovskites lie in a borderline stability regime, which is consistent with the small structural distortions obtained from our DFT calculations.

TABLE S1: Tolerance factor proposed by Bartel et al. to better estimate the peovskite stability.

Material	Tolerance Factor (τ)
Sc	4.23
Ti	4.24
V	4.23
Cr	4.23
Mn	4.23
Fe	4.22
Co	4.22
Ni	4.21

C. Orbital Overlaps Calculation

To quantify the energetic positions and hybridization of the transition-metal d states, orbital centroids were estimated from the projected density of states (PDOS). The centroid energy of an orbital manifold was calculated as the first moment of the corresponding PDOS within the energy window:

$$E_{\text{cent}} = \frac{\int_{E_{\min}}^{E_{\max}} E\, D(E)\, dE}{\int_{E_{\min}}^{E_{\max}} D(E)\, dE}, \tag{S4}$$

where $D(E)$ is the orbital-resolved PDOS, with $E_{\min}$ and $E_{\max}$ being the energy window of the concerned PDOS. The e_g manifold was obtained by summing the d_{z^2} and $d_{x^2-y^2}$ PDOS contributions, while the t_{2g} manifold was obtained from the sum of the d_{xy}, d_{xz}, and d_{yz} orbitals. For spin-polarized systems, the majority-spin channel was used for the overlap analysis, whereas the crystal-field splitting was determined from the centroids of the corresponding orbital manifolds.

The degree of hybridization between the TM-d and I-p states was quantified using the normalzied overlap integral of their PDOS:

$$O_{AB} = \frac{\int_{E_{\min}}^{E_{\max}} D_A(E) D_B(E)\, dE}{\sqrt{\int_{E_{\min}}^{E_{\max}} D_A^2(E)\, dE \int_{E_{\min}}^{E_{\max}} D_B^2(E)\, dE}}, \tag{S5}$$

where $D_A(E)$ and $D_B(E)$ represent the PDOS of the two orbital manifolds being compared. All numerical integrations were performed using the trapezoidal rule. The complete analysis script used to calculate the orbital centroids and overlap integrals has been made available in the accompanying Zenodo repository to ensure full reproducibility of the analysis.

D. DFT Parameters

The parameters and thresholds used in DFT calculations for all systems calculations are given in Table S2. The "mv" smearing is used.

TABLE S2: Parameters used in DFT calculations.

Parameter	Value
ecutwfc	40
ecutrho	320
etot_conv_thr	8.0×10^{-3}
forc_conv_thr	1.0×10^{-3}
conv_thr	1.60×10^{-5}
k-points	4 4 4

E. Hubbard Parameters

Hubbard parameters used for transition metals in DFT calculations are given in the table S3 below:

TABLE S3: Hubbard parameter values used in the study for transition metals. Most of the parameters are taken from materials project (MP) that were available there.

Material	Hubbard Parameter	Ref
Sc	7	[2]
Ti	3.5	[3]
V	3.25	[4]
Cr	3.7	[4]
Mn	3.9	[4]
Fe	5.3	[4]
Co	3.32	[4]
Ni	6.2	[4]

S2. RESULTS AND DISCUSSION

A. Stability Criteria and Structural Properties

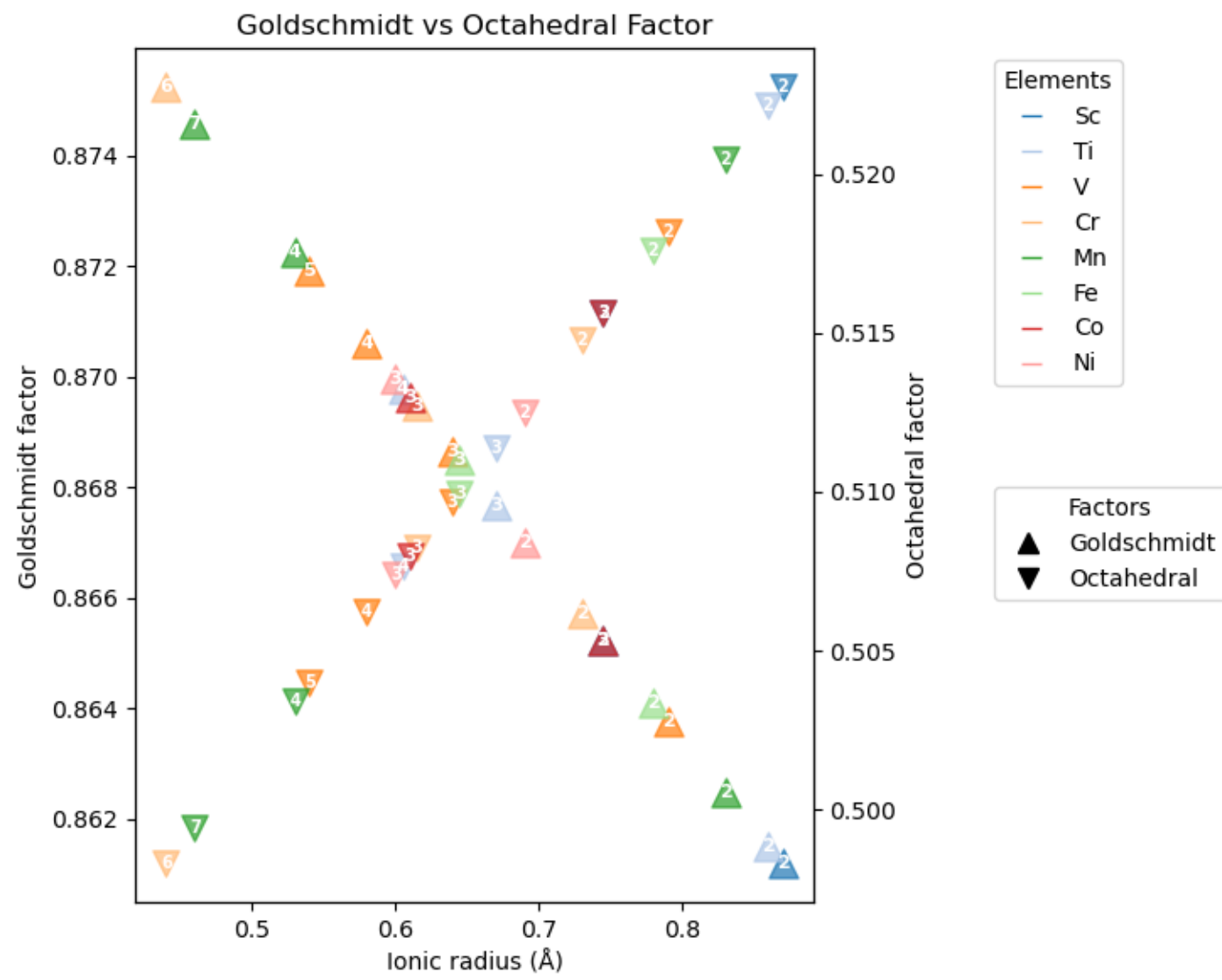

FIG. S1: Goldschmidt tolerance(octahedral) factor of all systems showing the linearly increasing(decreasing) trend with the size of ionic radii. All the stable defects with their lowest oxidation states exist in the ionic radii range of 0.69-0.86 Å.

TABLE S4: Structural properties of defected structures.

Material	Oxidation state	Ionic radius	Goldschmidt factor (t)	Octahedral factor (μ)
$CsPbI_3$	-	-	0.85	0.54
Sc_{Pb}	+2	0.83	0.86	0.52
	+3	0.74	0.87	0.52
Ti_{Pb}	+2	0.86	0.86	0.52
	+3	0.67	0.87	0.51
	+4	0.61	0.87	0.51
V_{Pb}	+2	0.79	0.86	0.52
	+3	0.64	0.87	0.51
	+4	0.58	0.87	0.51
	+5	0.54	0.87	0.50
Cr_{Pb}	+2	0.8	0.86	0.52
	+3	0.6	0.87	0.51
	+6	0.44	0.88	0.49
Mn_{Pb}	+2	0.83	0.86	0.52
	+4	0.53	0.87	0.50
	+7	0.46	0.87	0.49
Fe_{Pb}	+2	0.78	0.86	0.52
	+3	0.65	0.87	0.51
Co_{Pb}	+2	0.745	0.87	0.52
	+3	0.61	0.87	0.51
Ni_{Pb}	+2	0.69	0.87	0.51
	+3	0.6	0.87	0.50

B. Defect Formation Energies

TABLE S5: Growth conditions in which defect is more stable.

Stability region	Dopant	Oxidation states
I-rich	V	+2, +3, +4, +5
	Cr	+2, +3, +6
	Mn	+2, +4, +7
	Fe	+2, +3
	Co	+2, +3
	Ni	+2, +3
I-poor	Sc_{Pb}	+2, +3
	Ti_{Pb}	+2, +3, +4

Fig. S2 shows the trend of formation energies w.r.t ionic radii which suggest the kind of exponential decay in DFEs with increasing oxidation states. Also the lowest oxidation states of each defect creates most stable defect.

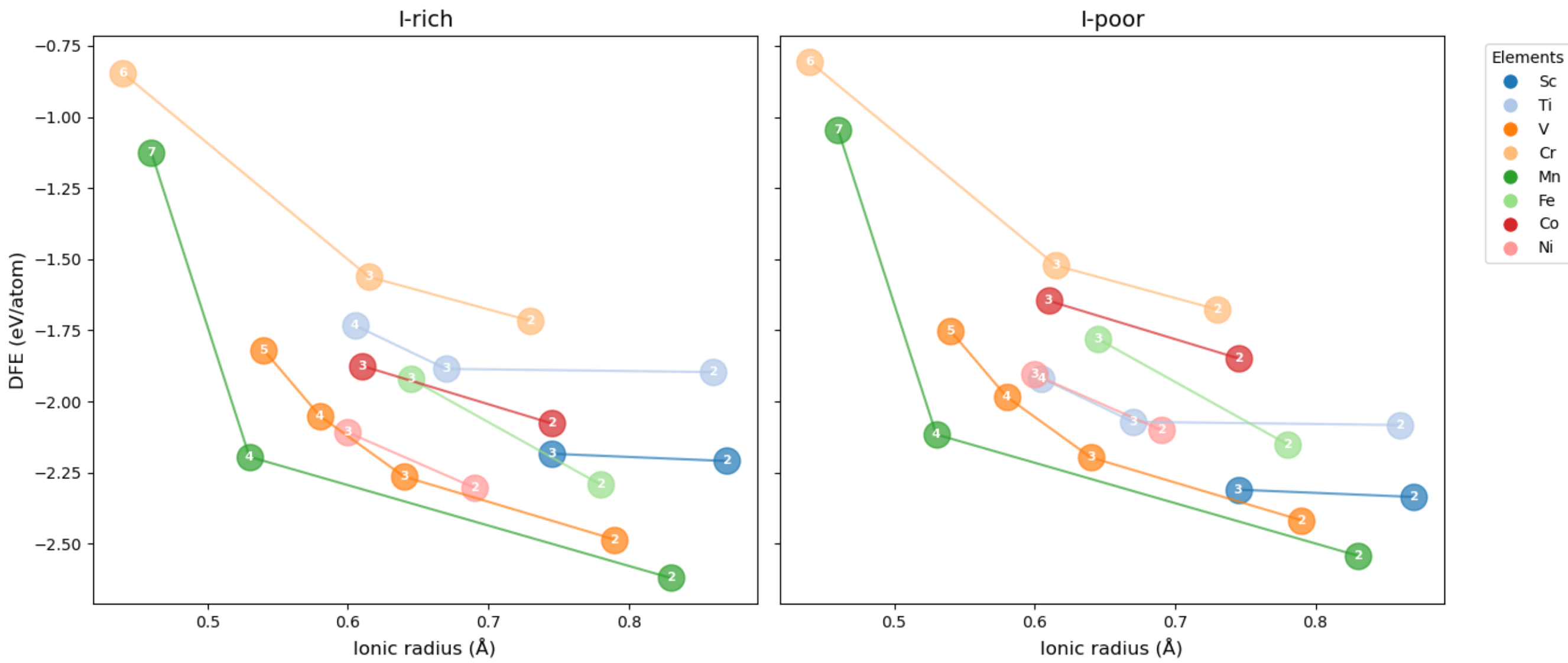

FIG. S2: DFE vs ionic radii in both I-rich and poor regions showing the DFE trend moving toward stability with decreasing oxidation state.

C. Stability Analysis

Table S6 shows that the TM-I bondlengths reduced in comaprison with the Pb-I bondlength in pristine $CsPbI_3$ which in result elongate the Pb-I bondlength adjacent to TM-I (with I common) and introduce local distortions in structure. These local distortions are given in Fig. S3 where effective coordination number (ECN) and bond length distortion shows the directl relation with TM-I bondlength.

TABLE S6: Octahedral bondlengths for pristine $CsPbI_3$ and doped structure showing the reduced values after doping.

Material	Pb-Halide bondlength	Dopant-Halide bondlength	Overlap e_g - I_p	Overlap t_2g - I_p
$CsPbI_3$	3.15	-	-	-
Sc_{Pb}	3.23	3.07	0.24	0.04
Ti_{Pb}	3.32	2.98	0.24	0.07
V_{Pb}	3.32	2.98	0.14	0.81
Cr_{Pb}	3.40	2.90	0.30	0.09
Mn_{Pb}	3.30	3.00	0.06	0.03
Fe_{Pb}	3.32	2.98	0.05	0.01
Co_{Pb}	3.36	2.94	0.11	0.10
Ni_{Pb}	3.35	2.95	0.10	0.05

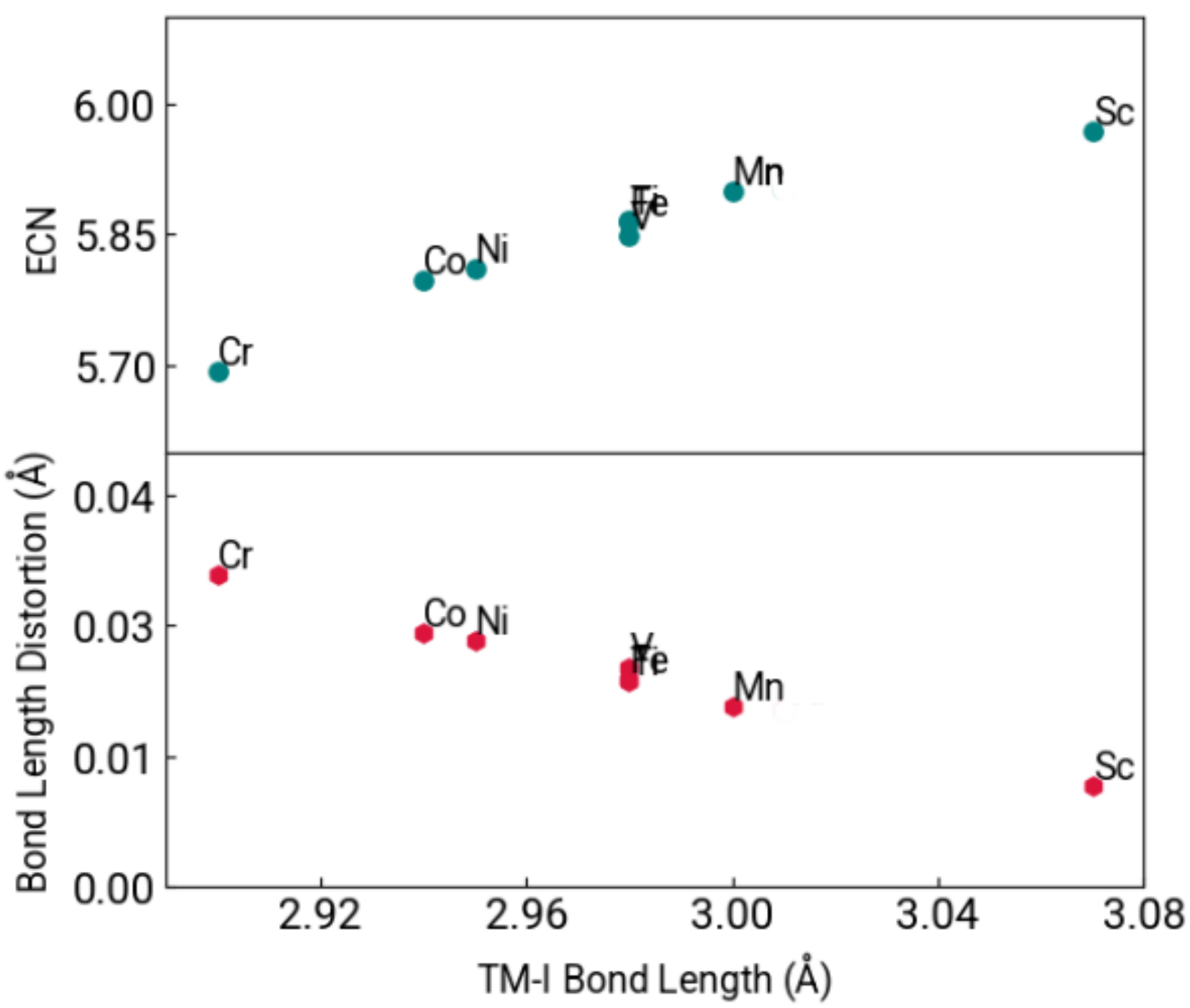

FIG. S3: Distortion parameters in the adjacent Pb octahedra: (top) Effective coordination number and (bottom) Bond length distortion .

[1] V. M. Goldschmidt, Die Gesetze der Krystallochemie, Naturwissenschaften 14 (21) (1926) 477–485. doi:10.1007/BF01507527.
URL https://doi.org/10.1007/BF01507527

[2] H. Ullah, S. Yasin, K. Safeen, A. Younus, Z. M. El-Bahy, A. Safeen, S. N. Abdou, M. M. Ibrahim, First principle study of scandium-based novel ternary half heusler scxge (x= mn and fe) alloys: insight into the spin-polarized structural, electronic, and magnetic properties, RSC advances 14 (19) (2024) 13605–13617.

[3] M. R. Preciado, A. Kassiba, A. Morales-Acevedo, M. Makowska-Janusik, Vibrational and electronic peculiarities of nitio 3 nanostructures inferred from first principle calculations, RSC Advances 5 (23) (2015) 17396–17404.

[4] A. Jain, S. Ong, G. Hautier, W. Chen, W. Richards, S. Dacek, S. Cholia, D. Gunter, D. Skinner, G. Ceder, et al., The materials project: a materials genome approach to accelerating materials innovation. apl mater 1: 011002 (2013).
